# Changing the cause of liver cirrhosis from hepatitis B virus to fatty liver in Iranian patients 

**Published:** 2017

**Authors:** Behzad Hatami, Sara Ashtari, Afsaneh Sharifian, Hamideh Rahmani Seraji, Elmira khalili, Yasin Hatami, Mohammad Reza Zali

**Affiliations:** 1 *Basic and Molecular Epidemiology of Gastrointestinal Disorders Research Center, Research Institute for Gastroenterology and Liver Diseases, Shahid Beheshti University of Medical Sciences, Tehran, Iran.*; 2 *Gastroenterology and Liver Diseases Research Center, Research Institute for Gastroenterology and Liver Diseases, Shahid Beheshti University of Medical Sciences, Tehran, Iran.*; 3 *Foodborne and Waterborne Diseases Research Center, Research Institute for Gastroenterology and Liver Diseases, Shahid Beheshti University of Medical Sciences, Tehran, Iran *

**Keywords:** Liver cirrhosis, Etiology, Epidemiology, Iran

## Abstract

**Aim:**

The objective of this study was to determine the epidemiologic characteristics of patients with liver cirrhosis.

**Background:**

Liver cirrhosis is an end-stage condition of chronic liver disease. Liver disease is an important cause of morbidity and mortality worldwide.

**Methods:**

In this cross-sectional study, demographic and epidemiologic characteristics of 203 patients with liver cirrhosis who were admitted in Taleghani Hospital over a period of two years were determined.

**Results:**

A total of 203 patients with liver cirrhosis consisted of 136 (67%) males and 67 (33%) females and the mean age was 53.7±15.2 years. No etiology were found in (59.6%) cirrhotic patients; therefore, they were labeled as cryptogenic cirrhosis, but according to clinical evidence and ultrasonography findings, (29.7%) of these patients were probable NAFLD. The other causes of liver cirrhosis in this study were HBV (20.2%), HCV (11.8%) and autoimmune hepatitis (AIH) (8.4%), respectively. Esophageal varices were present in (41.9%), ascites in (36.5%), variceal bleeding in (8.9%), encephalopathy in (7.4%) and spontaneous bacterial peritonitis (SBP) in (5.4%) of patients. When cirrhotic patients were grouped according to Child-Pugh classification, 26.1%, 54.7% and 19.2% were in classes A, B and C respectively. The mean MELD score was 16.16±7.7.

**Conclusion:**

In this study we found that the leading etiology of cirrhosis is cryptogenic cirrhosis 59.6% (in all age groups) and followed by HBV. Noteworthy, according to the clinical and ultrasonography findings, 29.7% of patients who were labeled as cryptogenic cirrhosis were consistent with NAFLD.

## Introduction

 Liver cirrhosis and its complications are major clinical problems that can cause a significant disability and mortality (1, 2). It is the most severe form of liver diseases and is characterized by the development of different nodes due to various liver diseases (3, 4). Epidemiologic factors of liver cirrhosis differ geographically and there are differences between ethnic groups (5). Several causes contribute to the growth of cirrhosis; the hepatitis B and C virus (HBV, HCV), alcohol consumption, non-alcoholic steatohepatitis (NASH), autoimmune hepatitis (AIH), and Wilson’s disease (6-9). In the United States, most European countries and Japan, HCV and alcohol are the most common causes of cirrhosis, whereas HBV-related liver cirrhosis is predominant in most Asian-Pacific and African countries (10-12).

Portal hypertension, ascites, gastroesophageal variceal bleeding, hepatic encephalopathy and spontaneous bacterial peritonitis (SBP) are common complications of cirrhosis (13, 14). Esophageal varices have great clinical impact, with a mortality risk of 17%–42% per bleeding episode (15). Ascites, an important complication of advanced cirrhosis and severe portal hypertension, is sometimes refractory to treatment and at times becomes complicated by (SBP) and hepatorenal syndrome. Hepatic encephalopathy is another complication with a mortality rate of about 30% (16). About 15% of patients with cirrhosis ultimately develop hepatocellular carcinoma (HCC) (17). The mortality rate of HCC associated with cirrhosis is increasing in most developed countries, while mortality due to non-HCC complications from cirrhosis is decreasing (18). Survival data from the United Network of Organ Sharing (UNOS) indicates a 1 year survival of 83%, a 5 year survival of 70% and an 8 year survival of 61% for cirrhotic patients (19). The ultimate treatment for cirrhosis and end stage of liver disease is liver transplantation.

Determining the cause of liver cirrhosis is very important. This can predict complications and the direct decisions for treatment and preventive measures for health care. However, only a few epidemiological studies have been conducted on cirrhosis in Iran. In this study we determined the epidemiologic characteristics of patients with liver cirrhosis at Tehran’s Taleghani Hospital within a period of two years from 2013 to 2015. 

## Methods

This is a cross-sectional study performed on cirrhotic patients who were admitted to the gastroenterology department of Taleghani Hospital, a referral teaching hospital in Tehran, Iran, from October 2013 to October 2015 and consist of 203 patients. Cirrhosis is a pathologic diagnosis, but because of coagulopathy in these patients, biopsy is rarely done and diagnosis is usually based on indirect stigmata of the disease. In this study, cirrhosis was diagnosed according to the clinical, laboratory and radiological criteria. Demographic data of the patients including age and sex were recorded according to a questionnaire specifically designed for this purpose. The etiology of cirrhosis was identified according to clinical findings and laboratory tests. Serologic tests were done for viral hepatitis (HBsAg, anti-HBc Ab, anti-HCV Ab) in all cases. In negative cases, HBV DNA PCR and HCV RNA PCR were done to rule out the occult forms of viral infection. In patients with no viral infections, Wilson disease, autoimmune hepatitis and hemochromatosis were ruled out with serum copper and ceruloplasmin, iron profile study (serum Iron (SI), total iron binding capacity (TIBC), Ferritin, SI/TIBC index), antinuclear antibodies (ANA), anti-smooth muscle antibodies (ASMA), anti-liver-kidney microsome-1(ALKM-1) and protein serum electrophoresis, respectively. If above etiologies were ruled out, Anti tissue transglutaminase IgA (Anti TTG IgA) and serum alpha1 antitrypsin were measured to rule out Celiac disease and alpha1 antitrypsin deficiency, respectively. If no etiology was found, the patients were labeled as cryptogenic cirrhosis. For metabolic (fasting blood sugar (FBS)); total cholesterol, triglyceride, low-density cholesterol (LDL-C) and high-density cholesterol (HDL-C) were determined. Severity of liver cirrhosis was evaluated by Child-Pugh classification and Model for End-stage Liver Disease (MELD) scores (20, 21) . For this purpose, complete blood count (CBC), serum albumin level, serum bilirubin level, serum BUN and creatinine level, prothrombin time (PT) and international normalized ratio (INR) were enrolled. Presence of ascites, hepatic encephalopathy, HCC and history of liver transplantation were recorded in the questionnaire. CHILD and MELD scores calculated according to standard formulas and analyzed using the www.unos.org website (22). 

Abdominal ultrasonography was done for all the patients to evaluate parenchyma and size of the liver and spleen, and detect presence of ascites and liver lesions. If there was a liver lesion, it was evaluated according to the guidelines to rule out HCC. In case of ascites, we performed paracentesis to look for spontaneous bacterial peritonitis (SBP). If there was an increased level of serum creatinine (> 1.5 mg/dL) in patients' lab exams, they were evaluated to rule out hepatorenal syndrome (23-25). All patients underwent upper endoscopy to detect and evaluate gastro-esophageal varices.

Data were entered and analyzed using statistical package for social sciences (SPSS) for windows version 21 software (SPSS Inc., Chicago, IL, USA). Descriptive statistics and frequency distribution such as mean, standard deviation and percentage were employed. A ***p***-value of <0.05 was considered significant. 

## Results

A total of 203 cirrhotic patients were enrolled in this study. They consisted of 136 (67%) males and 67 (33%) females (male to female ratio was 2:1), with a mean age of 53.7±15.2 years (range: 14-86 years). The age ranges further categorized into 4 groups (table1). 

The results based on different age groups in this study shows that cryptogenic cirrhosis has the highest rate in all age groups. After cryptogenic cirrhosis, HBV infection was the most common cause of cirrhosis in patients over 60 years of age. HCV was more common in patients between 40-60 years old and AIH was the predominant cause of cirrhosis in patients between 20-40 years old (table2). According to clinical evidence and ultrasonography findings, 29.7% of patients with cryptogenic cirrhosis were probable NAFLD. Twenty six (72.2%) of them were male, while 10 (27.8%) were female. Frequency of probable NAFLD in age groups of <20, 20-40, 40-60 and >60 were 4 (11.1%), 4 (16.7%), 14 (38.9 %) and 12 (33.3%) respectively. 

**Table1:** Age group and Etiology of patients by sex

Variables		Male (%)	Female (%)	Total (%)
Age groups	<20	6 (7.6)	0 (0)	6 (3.0)
	20-40	20 (14.7)	12 (17.9)	32 (15.8)
	40-60	58 (42.6)	27 (40.3)	85 (41.9)
	>60	52 (38.2)	28 (41.8)	80 (39.4)
Total		136 (100)	67(100)	203 (100)
Etiology	HBV#	31 (22.8)	10 (14.9)	41 (20.2)
	HCV¢	21 (15.4)	3 (4.5)	42 (11.8)
	AIH*	4 (2.9)	13 (19.4)	17 (8.4)
	Cryptogenic	80 (58.8)	41 (61.2)	121 (59.6)
Total		136 (100)	67 (100)	203 (100)

# Hepatitis B virus

¢ Hepatitis C virus

* Autoimmune Hepatitis

**Table 2:** Etiology of liver cirrhosis by age group

Etiology	Age groups				Total
	<20	20-40	40-60	>60	
HBV#	0 (0)	4 (12.5)	14 (16.5)	23 (28.8)	41 (20.2)
HCV¢	0 (0)	2 (6.3)	17 (20)	5 (6.3)	24 (11.8)
AIH*	0 (0)	5 (15.6)	10 (11.8)	2 (2.5)	17 (8.4)
Cryptogenic	6 (100)	21 (65.6)	44 (51.8)	50 (62.5)	121 (59.6)
Total	6 (100)	32 (100)	85 (100)	80 (100)	203 (100)

# Hepatitis B virus

¢ Hepatitis C virus

* Autoimmune Hepatitis

The most frequent complications of liver cirrhosis in present study were esophageal varices (41.9%), ascites (36.5%), variceal bleeding (8.9%), encephalopathy (7.4%), and SBP (5.4%). Liver-related mortality in one-year follow up occurred in 24 (11.8%) patients (table 3).

**Table 3.** Complication of liver cirrhosis by age, sex, etiology and CHILD class groups

Variables		Complication				
		GEV*	Ascites	VB #	ECP ∞	SBP ≠
Sex	Male Female	65 (76.5) 20 (23.5)	53 (71.6) 21 (28.4)	14 (77.8) 4 (22.2)	12 (80) 3 (20)	10 (90.9) 1 (9.1)
Total		85 (100)	74 (100)	18 (100)	15 (100)	11 (100)
Age Groups	<20 20-40 40-60 >60	3 (3.5) 7 (8.2) 33 (38.8) 42 (49.4)	2 (2.7) 9 (12.2) 27 (36.5) 36 (48.6)	1 (5.6) 1 (5.6) 8 (44.4) 8 (44.4)	0 (0) 1 (6.7) 8 (53.3) 5 (40)	1 (9.1) 1 (9.1) 5 (45.5) 4 (36.4)
Total		85 (100)	74 (100)	18 (100)	15 (100)	11 (100)
Etiology	HBV# HCV¢ AIH* Cryptogenic	23 (27.1) 13 (15.3) 3 (3.5) 46 (54.1)	15 (20.3) 11 (14.9) 2 (2.7) 46 (62.2)	3 (16.7) 3 (16.7) 0 (0) 12 (66.6)	3 (20) 1 (6.7) 0 (0) 11 (73.3)	2 (18.2) 2 (18.2) 0 (0) 7 (63.6)
Total		85 (100)	74 (100)	18 (100)	15 (100)	11 (100)
Child Class	A B C	23 (27.1) 43 (50.6) 19 (22.4)	8 (10.8) 38 (51.4) 28 (37.8)	5 (27.8) 8 (44.4) 5 (27.8)	0 (0) 3 (20) 12 (80)	1 (9.1) 5 (45.5) 5 (45.5)
Total		85 (100)	74 (100)	18 (100)	15 (100)	11 (100)

* Gastroesophageal Varices,

# Variceal Bleeding,

∞ Encephalopathy,

≠ Spontaneous Bacterial Peritonitis

**Table 4:** Child Class of patients by etiology and age group

Variables		Child Class			Total
		A (%)	B (%)	C (%)	
Etiology	HBV#	14 (26.4)	20 (18)	7 (17.9)	41 (20.2)
	HCV¢	6 (11.3)	10 (9)	8 (20.5)	24 (11.8)
	AIH*	7 (13.2)	7 (6.3)	3 (7.7)	17 (8.4)
	Cryptogenic	26 (49.1)	74 (66.7)	21 (53.8)	121 (59.6)
Total		53 (100)	111 (100)	39 (100)	203 (100)
Age Groups	<20	0 (0)	5 (4.5)	1 (2.6)	6 (3.0)
	20-40	8 (15.1)	19 (17.1)	5 (12.8)	32 (15.8)
	40-60	25 (47.2)	44 (39.6)	16 (41)	85 (41.9)
	>60	20 (37.7)	43 (38.7)	17 (43.6)	80 (39.4)
Total		53 (100)	111 (100)	39 (100)	203 (100)

# Hepatitis B virus

¢ Hepatitis C virus

* Autoimmune Hepatitis

**Table 5:** MELD scores based on etiology and age of patients

Variables			MELD Score				Total
		<10	10-20	20-30	30-40	>40	
Etiology	HBV#	8 (16)	23 (21.5)	8 (22.2)	1 (16.7)	1 (25)	41 (20.2)
	HCV¢	6 (12)	14 (13.1)	4 (11.1)	0 (0)	0 (0)	24 (11.8)
	AIH*	3 (6)	10 (9.3)	2 (5.6)	1 (16.7)	1 (25)	17 (8.4)
	Cryptogenic	33 (66)	60 (56.1)	22 (61)	4 (66.7)	2 (50)	121 (59.6)
Total		50 (100)	107 (100)	36 (100)	6 (100)	4 (100)	203 (100)
Age Groups	<20	1 (2)	4 (3.7)	1 (2.8)	0 (0)	0 (0)	6 (3)
	20-40	6 (12)	16 (15)	8 (22.2)	1 (16.7)	1 (25)	32 (15.8)
	40-60	3 (60)	40 (37.4)	12 (33.3)	3 (50)	0 (0)	85 (41.9)
	>60	13 (26)	47 (43.9)	15 (41.7)	2 (33.3)	3 (75)	80 (39.4)
Total		50 (100)	107 (100)	36 (100)	6 (100)	4 (100)	203 (100)

# Hepatitis B virus

¢ Hepatitis C virus

* Autoimmune Hepatitis

From 203 participants in this study, only 2 (1%) patients underwent liver transplant and 36 (17.7%) of them underwent band-ligation for esophageal varices. According to the Child-Pugh classification, 26.1% of patients were classified as A class, 54.7% as B class and 19.2% as C class (table 4). In our study the mean MELD scores was 16.16±7.7 (range: 6-50) (table 5).

## Discussion

Liver cirrhosis is the most common hepatic cause for hospitalization in gastroenterology and hepatology wards, as well as the third cause of death in Iran (26). Due to lack of national registration system, there is limited information on the cause and clinical features of cirrhosis in Iran. In this study, the epidemiological characteristics of 203 patients with cirrhosis in Taleghani Hospital of Tehran during two years have been determined.

No etiology were found in (59.6%) cirrhotic patients in this study; therefore, they were labeled as cryptogenic cirrhosis. However, based on clinical and laboratory evidence and ultrasonography findings, 36 patients who labeled as cryptogenic cirrhosis, were probable non-alcoholic fatty liver (NAFLD). Several explanations may be offered as possible underlying etiologies, but today NASH is recognized as the most common cause of cryptogenic cirrhosis (27). Obesity and non-insulin-dependent diabetes mellitus are the two most common conditions associated with NASH (28), which are frequently asymptomatic (29) and can progress silently to cirrhosis with loss of definitive histological features. Percutaneous liver biopsy is high risk in cirrhotic patients and considering that fatty liver is asymptomatic, make the diagnosis of NASH really difficult. Therefore, most of the patients with NASH are identified as cryptogenic cirrhosis. 

The results based on different age groups in this study show that cryptogenic cirrhosis has the highest rate in all age groups. After cryptogenic cirrhosis, AIH (15.6%), HCV infection (20%) and HBV infection (28.8%) were the common causes of cirrhosis in patients in 20-40, 40-60 and over 60 year age groups, respectively. Based on previous studies in Iran and Asia, HBV was reported to be the leading etiology of liver cirrhosis (30, 31). But according to recent studies, it seems that the rate of HBV-related cirrhosis is declining and the rate of NAFLD-related cirrhosis is arising (32). After implementation of the HBV National Vaccination Program for all newborns and high risk groups since 1993, along with availability of medical experts and anti-viral medications in Iran, the prevalence of the virus decreased dramatically in young population, but it is still high at old age (12, 33). In our study, HBV was the common cause of cirrhosis in patients over 60 years old, as well. On the other hand, the prevalence of obesity, Type II diabetes and metabolic syndrome (risk factors of fatty liver) are increasing worldwide (8, 34, 35) . One of the limitations of our study is that we did not perform liver biopsy for our patients (due to risk of bleeding), but based on clinical and laboratory evidence and ultrasonography reports, at least 17.7% of our patients (29.7% of cryptogenic cirrhosis patients) have probable NAFLD and probably NASH. The important point is that most of these patients were older than 40 years old (72.2%) and NAFLD was probably the most common cause of cirrhosis in patients under 40 years of age. 

Patients with cirrhosis are susceptible to variety of complications and their life expectancy is significantly reduced. The occurrence of complications increases the mortality rate in patients with liver cirrhosis. Therefore, early diagnosis and treatment of cirrhosis complications are important (36). The most frequent complication of liver cirrhosis in present study was varices (41.9%) which is completely similar to world gastroenterology organization Global Guidelines that reported the frequency of varices varies from 30% to 70% in patients with cirrhosis (37). Ascites is another major complication of cirrhosis (38), it is reported in (50%) of patients over 10 years of follow up by Gines P et al. (39). The development of ascites is an important landmark in the natural history of cirrhosis as it is associated with a (50%) mortality over two years and signifies the need to consider liver transplantation as a therapeutic option (40, 41). Ascites in this study presented in (36.5%) of patients, which divided in two groups as mild and severe; 71.6% and 28.4% respectively. Hajiani et al. in 2012 reported that in his study, ascites was present in (32%) of patients (5). Spontaneous bacterial peritonitis (SBP) is defined as an ascites fluid infection without an evident intra-abdominal surgically-treatable source; it primarily occurs in patients with advanced cirrhosis. SBP is an important complication of cirrhosis and if it is not diagnosed and treated early, shock ensues, followed rapidly by multisystem organ failure. Survival is unlikely in patients who develop shock prior to initiation of empiric antibiotics. The incidence of SBP in cirrhotic patients admitted to hospital has been estimated to be 10-30% (42-44) . Nevertheless, recent studies demonstrate that the incidence of SBP is decreasing (45) . Results of this study also show decreased rate of SBP (5.4%). This can be due to wide use of antibiotics for SBP prophylaxis. Hajiani et al. reported the frequency of 8% and 1% for variceal hemorrhage and encephalopathy, respectively (5). However, in our study frequency of variceal bleeding and encephalopathy were (8.9% and 7.4%) respectively. Taleghani Hospital is a tertiary and referral center and the higher frequency of these grave complications (which need hospitalization and expert management) can be due to this fact.

The Child-Pugh classification is a simple convenient prognostic measure in patients with liver cirrhosis that has been repeatedly shown to be useful in this assessment. Hajiani et al. (5) reported in his study (19%) as A class, (30%) as B class and (51%) as C, and also in the study of liver cirrhosis over 10 years in China reported (31.2%) as A class, (41.7%) as B class and (27.1%) as C class (29). In the current study, (26.1%) were assigned to A class, (54.7%) as B class and (19.2%) in C class. Comparing our results to Hajianis’, most of our patients were in B class. 

Recently, the MELD score has been shown to be superior to the Child-Pugh score in ranking patients according to the severity of liver disease and risk of mortality. The MELD score is considered to be more reproducible than the Child-Pugh score because it does not include subjective variables such as ascites and encephalopathy (21). The mean of MELD scores in this study was 16.16±7.7 that is comparable with 15.3±7.2 in the Wang et al. study (29).

In summary, in this study we found that 59.6% of patients had cryptogenic cirrhosis. According to clinical and laboratory evidence and ultrasonography findings, 36 (29.7%) of patients with cryptogenic cirrhosis were probable NAFLD. These results will suggest that Fatty liver should be considered as one of the main causes of cirrhosis (especially in young patients). The prevalence of obesity, Type II diabetes and metabolic syndrome (risk factors of fatty liver) is increasing worldwide. Results of this study also show an increase number of cirrhosis due to fatty liver, especially in patients older than 40 years old. Therefore, early detection, appropriate treatment and also care programs with public awareness and training about NAFLD can be considered as an effective step in controlling and reducing the incidence of liver cirrhosis. On the other hand, as regards to worldwide declining pattern of HBV prevalence due to prevention programs (vaccination), prevalence of HBV cirrhosis has declined in younger patients. Also new treatments for HCV are very effective and have high cure rates; therefore, it seems that in near future, HCV will not be a major etiology for cirrhosis. 

Further investigations with larger sample numbers are necessary to determine the causes and various clinical complications. We have to implement a prospective study to follow these cases for a longer period to define the outcome. Since limited data is available about cirrhosis and frequency of its complications in Iran, the present study is helpful for better management of liver cirrhosis and its complications.
